# Malnutrition, morbidity and infection in the informal settlements of Nairobi, Kenya: an epidemiological study

**DOI:** 10.1186/s13052-019-0607-0

**Published:** 2019-01-14

**Authors:** Maria Vittoria De Vita, Carlo Scolfaro, Bruna Santini, Antonella Lezo, Federico Gobbi, Dora Buonfrate, Elizabeth W. Kimani-Murage, Teresiah Macharia, Milka Wanjohi, Jacopo Mattia Rovarini, Gianfranco Morino

**Affiliations:** 1 Amici del Mondo - World Friends Onlus / Ruaraka Uhai Neema Hospital, P.O. Box 39433-00623, Nairobi, Kenya; 2 Department of Pediatrics - Infectious Diseases Unit - Regina Margherita Children’s Hospital, University of Turin, Turin, Italy; 3 Division of Nutrition, Regina Margherita Children’s Hospital, University of Turin, Turin, Italy; 4 Centre for Tropical Diseases, IRCCS-Ospedale Sacro Cuore don Calabria, Negrar, 37024 Verona, Italy; 5 African Population and Health Research Centre, APHRC Campus, P.O. Box 10787-00100, Nairobi, Kenya; 6 World Friends (Kenya) @ Ruaraka Uhai Neema Hospital, Off Thika Rd, opp. Safari Park Hotel, P.O. Box 29433-00623, Nairobi, Kenya

**Keywords:** Malnutrition, Infection, WASH, Informal settlements, Kenya

## Abstract

**Background:**

Malnutrition constitutes one of the major public health challenges throughout the developing world. Urban poverty and malnutrition have been on the rise, with an increased rate of morbidity. We herein explore the relationship between infections and nutritional status and the related association with hygienic conditions as risk of infection in children residing in the slums of Nairobi.

**Methods:**

Case-control study based on a secondary analysis of quantitative data collected from a cluster randomized trial carried out in two slums of Nairobi. The following information about resident children were selected: babies’ anthropometric measurements, related life conditions, data on infant-feeding practices, food security, hygiene, immunization coverage and morbidity were collected and updated with structured questionnaires until 12 months of life. Prevalence of malnutrition was calculated, then both bivariate and multivariate analysis were used to explore the relationship between malnutrition and its determinants.

**Results:**

The study involved a total of 1119 babies registered at birth (51.28% male and 48.03% female infants). Overall the prevalence of malnutrition was high, with 26.3% of the children being stunted, 6.3% wasted and 13.16% underweight. Prevalence of wasting was higher in the first months of life, while in older children more case of stunting and underweight were captured. Wasted infants were significantly associated with common childhood illnesses: with cough and rapid breathing as well as with diarrhea (*p*-value< 0.05). Stunting was associated with hygienic conditions (p-value< 0.05 in households that did not perform any water treatment and for children that had a toilet within the house compound), immunization program and low-birth-weight. Moreover, regression analysis showed that significant determinants of stunting were sex and feeding practices. Underweight was significantly associated with socio-demographic factors.

**Conclusions:**

In the specific environment where the study was conducted acute malnutrition is correlated with acute infections, while chronic malnutrition is more influenced by WASH conditions. Therefore, our findings suggest that one cannot separate infection and its risk factors as determinants of the whole malnutrition burden.

## Background

Malnutrition is a major global public health challenge. Worldwide, it is estimated that 165 million children under 5 years of age are stunted, most of them in Africa and South and Central Asia [1]. Undernutrition in childhood causes 3.1 million child deaths annually [2]; it is associated with 35% of deaths in children less than 5 years old [3]. Concurrently, the latest estimates show that half of the 10.6 million under five child deaths per year in the world has been ascribed to five infectious diseases (pneumonia, diarrhea, malaria, measles, and Acquired Immunodeficiency Syndrome) [4]. The relationship between infectious diseases and poor nutritional status has been long established. Malnutrition causes an exponential decrease of the chances of survival per exposure to disease, within any given population. Children with the worst anthropometric parameters present an elevated risk of death from a variety of severe infections including sepsis, meningitis, measles and tuberculosis. Moreover, non-fatal and subclinical infections may impair the process of growth: several studies report that 46–80% of all nutrition-related deaths are a consequence of mild to moderate malnutrition [5]; evidence indicates that malnourished children have a clear excess risk of infectious morbidity and mortality [6]. Less quantified is the inverse relationship, from infection to malnutrition, even if it is well known that infections alter nutrients intake, absorption, secretion, catabolism and consumption. This may result in a vicious cycle of infection and child malnutrition, with an inter-relation that clearly appears dynamic in nature.

The ever-increasing speed of the urbanization process raises new concerns about the spread of malnutrition in such settings. The rapid growth of the urban population in a context of inadequate urban planning led to the mushrooming of slums. As a result, the majority (56% in 2014) [7, 8] of urban residents in sub-Saharan Africa live in overcrowded shantytowns, characterized by very poor housing conditions, inadequate water and sanitation infrastructures and deficient health facilities [9]. In informal settlements, malnutrition is strongly associated with lack of food [10] and other forms of deprivation (assets and subjective poverty), with an elevated rate of morbidity [11]. In this environment, nutrient deficiencies and infections are influenced by several factors and child morbidity has similar determinants of malnutrition. As a result, there is a double burden of malnutrition and infections.

New evidence shows that inadequate sanitation, poor hygiene and lack of access to clean drinking water constrain linear growth in children [12]. Nutritionists are now collaborating with experts in the field known as Water, Sanitation and Hygiene (WASH), to improve sanitary conditions in countries afflicted by high stunting rates.

The risk factors and co-occurring issues for children living in the urban disadvantaged areas show that malnutrition is not only related to nutrient intake but to multiple factors. The rationale of this study is to assess the influence of infections and hygiene on nutritional status in children residing in two slums (Viwandani and Korogocho) of Nairobi, Kenya, one of the biggest cities in Sub-Saharan Africa.

Nairobi has more than 40 areas defined as slums and about 56% of Nairobi City County population lived in slums in 2014 [13]. The significant advantage in health and other social development indicators that urban areas have traditionally witnessed over rural areas are dwindling or even reversing [9, 14, 15].

## Methods

### Objective of the study

We herein explore the relationship between infections and nutritional status and the related association with hygienic conditions as risk of infection in children residing in above-mentioned slums.

### Specific objectives

1. To examine the relationship between malnutrition and the occurrence of common infections in children living in informal settlements in Nairobi (Kenya).
2. To examine the relationship between hygiene, water and sanitation conditions as a risk factor for infection and malnutrition in children living in informal settlements in Nairobi (Kenya).

### Study site

The MYICN (Maternal Infant and Young Child Nutrition) Intervention study, a cluster randomized controlled trial, was carried out in two slums of Nairobi, Kenya, i.e. Korogocho and Viwandani; here the African Population and Health Research Center (APHRC) runs the Nairobi Urban Health and Demographic Surveillance System (NUHDSS), covering close to 70,000 residents. The two slums are located about 7 km from each other and are densely populated with 63,318 and 52,583 inhabitants per square kilometer respectively [16].

The NUHDSS involves a systematic quarterly recording of vital demographic events, including births, deaths and migrations occurring among residents of all households in the area since 2003. Other data are also collected and updated regularly [17].

The informal settlements of Viwandani and Korogocho were selected as study sites, since data from available literature showed high malnutrition prevalence in these areas; with 45% of children aged < 5 years stunted and high under-five children mortality (79 deaths/1000 live births) [18] these settlements performed worse than other populations in Kenya, including those residing in rural settings and other areas of Nairobi, considering mortality, rate of infections and life conditions [9].

### Study design

Case-control study based on a secondary analysis of quantitative data collected by the antecedent MIYCN intervention study. The latter’s protocol has already been published [19, 20] together with the results of the intervention [21]. Therefore we herein only detail methods relevant to the specific research question of this paper.

### Study population & enrollment

The source population consisted in all pregnant women and their offspring living in the randomized Community Health Units (CUs, defined by the Kenya National Community Health Strategy) in Korogocho and Viwandani slums that fall within the NUHDSS area.

The *inclusion criteria* were as follows: all pregnant women aged between 12 and 49 years and their children,born between December 2012 and July 2014.

*Exclusion criteria* were as follows: women with a disability that made the administration of the questionnaires challenging (e.g. hearing, sight or mental impairment); women of reproductive age who had given birth before the recruitment started; disability in both mother and child that would significantly affect infant feeding (e.g. developmental problems); women who had a miscarriage or a still-birth; women who were lost to follow-up during pregnancy.

Recruitment of participants was conducted through routine NUHDSS rounds, whereby pregnancy registration is done for female residents in each household. This was complemented by case finding carried out by Community Health volunteers (CHVs) and informants to ensure high coverage.

While the reproductive age in most studies is usually defined as 15 to 49 years, girls aged 12 to 14 years were included because a substantial proportion (close to 10%) of adolescents in the study areas is sexually active before the age of 15 years [22].

### Data collection

Within the design of the MIYCN trial, which had focused on optimal maternal and infant feeding practices (as recommended by the WHO - World Health Organization), the population had been randomly divided into two groups: the intervention group and the control group. Both groups were involved in regular visits by CHVs, according to the specific needs of every age group [21]. In the intervention group, mothers were provided with age specific counseling and support on optimal child feeding and health, including breastfeeding initiation, exclusive breastfeeding (EBF), extended breastfeeding, complementary feeding, maternal nutrition, antenatal care including birth planning, health care seeking for delivery and post-natal services including immunization and general hygiene and child care. They also received information materials on MIYCN. The mothers in the intervention arm were visited at least monthly during pregnancy until gestation week no. 34, after which they were visited every week until they gave birth, and more frequently (as necessary) in the 1st month after giving birth (for support in initiating breastfeeding and sustaining EBF). They were then visited once a month until the 5th month, when they were visited fortnightly (to prepare them for introduction of complementary feeding) and monthly in the subsequent months until one year of age of the child. The control arm received standard care involving CHVs visits providing counselling on antenatal care, postnatal care including immunization and general hygiene in accordance with the guidance set forth by the community health strategy. The frequency of visits was defined by need, but generally about once a month per household, and usually more frequent around the time of birth. No specific schedule was given to them and CHVs in the control arm did not undergo any specific training on child feeding. However, mothers in the control arm also received information materials on MIYCN.

In the MIYCN Intervention study, babies’ anthropometric measurements, data on infant feeding practices, household characteristics, demographic factors, food security and hygiene, immunization coverage and morbidity were collected and updated (where relevant) every two months during follow-up visits in both groups (intervention and control). Data were captured using various researcher-administered questionnaires. With regards to the present study specific data have been selected from the data dictionary (where all information had been stored).

### Study variables

#### Dependent variable

***Malnutrition***, indicated by wasted, stunted and underweight children. Anthropometric measurements (weight, length) were taken according to standard procedures [23]. Prevalence of malnutrition in the population was generated and the related prevalence of stunting, underweight and wasting at different ages was also considered. For determination of underweight, stunting, and wasting, we calculated weight-for-age z-scores (WAZ), height-for-age z-scores (HAZ) and weight-for-height z-scores (WHZ), using the WHO 2007 growth standards [23, 24]. Stunting was determined as HAZ < − 2, underweight as WAZ < − 2 and wasting as WHZ < − 2 [25].

#### Independent variables

Independent variables were categorized into three groups.

1. ***Occurrence of infections (Morbidity)***

Concerning child health status, common childhood symptoms of illnesses occurring two weeks before the interview date were considered. These included: fever, cough, cough with rapid breathing, diarrhea and seizures. The five common childhood illnesses’ symptoms were counted as separate variables in the analysis, but also collectively as morbidity (general morbidity), when a child had at least a single episode of illness - regardless of the type - two weeks before the interview date.

2) ***WASH conditions***

Household Water, Sanitation and Hygiene (WASH) conditions were measured using data from a structured questionnaire addressing water, food and personal hygiene. One of the considered variables was whether any kind of water treatment had been in use at home; possible answers were: no treatment; filtered water; boiled water; water guard/aquatabs/other chemical treatment; sedimentation; UV rays or solar disinfection; sieved through cloth; others. Variables about habits of washing utensils for feeding babies, hand washing practice with soap (with specified frequency), presence of a toilet facility in or near the household were also investigated. With regards to sanitation, type of toilet facility used during the day and at night was categorized into two groups: own (own flush, traditional pit, Ventilated Improved Pit) and shared (shared flush, traditional pit, shared Ventilated Improved Pit; flush trench toilet; toilet without pit, working flush; no facility or bush and field, or flying toilet, and other toilet facility).

3) ***Control variables***

*Socio-economic and demographic variables:* the following were included: sex of the child; mother’s age, parity and occupation; marital status; religion; attained level of mother’s education.

*Birth Weight (BW):* BW was categorized into three groups: Low Birth Weight (LBW, less than 2.5 Kg), normal weight at birth (between 2.5 and 4.2 Kg) and overweight (more than 4.2 Kg at birth).

*Immunization coverage:* The variable on full vaccination among children according to the Kenyan Immunization Program was considered [26].

*Exclusive breastfeeding until six months:* Data were collected through the interviewer-administered questionnaire to the mother to determine if the child was still breastfeeding, and, if so, whether they had started feeding on other foods or fluids other than breast milk; when they were introduced to the other foods or fluids; and if they stopped breastfeeding, when they stopped [21].

*Complementary feeding practices:* this focused on complementary feeding practices with regard to WHO recommendations [27]. In the present study we focused only on: timely introduction of solid/semi-solid/soft foods; number of food groups consumed (at least four food groups consumed and less than four); minimum meal babies’ frequency and household food security situation.

*Household Food Security*: Household food security was defined using a modified Household Food Insecurity Access Scale (HFIAS) [28] .

### Data management and analysis

The WHO growth reference 2007 for children was used to generate anthropometric indices to assess the nutritional status of children [23, 25, 29]. The indices were expressed as standard deviation units from the median of the WHO child growth standards adopted in 2007. Bivariate analysis was conducted to evaluate the association between dependent and independent variables and to identify determinants of malnutrition in the study population. Odds Ratio (OR) and their 95% confidence intervals (CI) were estimated in order to show the magnitude of the association between independent variables and malnutrition. *P*-values of less than 0.05 were considered statistically significant.

All independent variables were analyzed initially in bivariate models and the variables that were significantly associated with the dependent variable were included in logistic regression models. Subsequent selection of variables fitted into the final models was based on statistical significance of *p*-value ≤0.25, as proposed by Hosmer and Lameshow [30], upon running univariable logistic regression with all the exploratory variables considered in the study. Adjustment for confounding factors were made for the associations observed between independent variables and dependent variables. Details on data collection procedures and other data collected are published [19]. Data management and analysis were carried out using STATA Version 13.

## Results

The study involved a total of 1119 children registered immediately after birth (51.28% male infants and 48.03% female infants).

### Nutritional status of the children

A total of 102 (9.10%) children were born with low birth weight (< 2.5 Kg); 993 (88.74%) of the children weighed between 2.5 Kg and 4.2 Kg and 24 (2.17%) weighed more than 4.2 Kg.

The prevalence of stunting among children under 12 months of age was 26.30%. The highest proportion of stunted children (31.40%) was observed between the age of 9 to < 12 months, with the lowest (22.06%) being observed for children below three months. Wasting for children under 12 months of age was 6.30%. Most cases of wasting were observed among children below three months (9.10%) with the lowest (3.97%) being observed between the ages of three to five months. The total prevalence of underweight was 13.16%, with the highest prevalence (16.97%) from 6 to the end of 8 months of age and the lowest (11.26%) for children aged from three to five months.

### Occurrence of infections

Among children under the age of 12 months, 9.35% had fever two weeks before the survey, with a trend of increased prevalence (10.38%) in the older infants. Amongst the total visited babies 14.98% had cough two weeks before the study visit, with the highest number of cases being observed in the group of babies aged between 3 and 6 months (20.26%). A proportion of 3.97% of all observations reported cough associated with rapid breathing at least once during the follow-up period two weeks before the interview date. Diarrhea occurred at least once in the two weeks preceding the study in 8.44% of the children visited, with increased prevalence in older infants (13.08% from 6 to 9 months, 14.39% from 9 to 12 months). The episodes of seizures were difficult to be recorded (especially due to poor awareness of the specific manifestation and the rare occurrence of the illness) and were reported as occurred only in the 0.35% of the visited babies. A total of 24.64% of all children were reported to have had at least one episode of either diarrhea, fever, cough, cough with rapid breathing or seizures (general morbidity) two weeks prior to the study visit. There was a higher prevalence of symptoms in older babies.

### The relationship between malnutrition and morbidity

There were no significant associations between any outcomes of malnutrition and general morbidity, as general occurrence of any kind of illness (*p*-value> 0.05). However, children with cough and rapid breathing had a two-fold higher likelihood of being wasted (OR = 2.13; 95% CI = 1.08, 4.22), (*p*-value = 0.03). Isolated cough was significantly associated (*p*-value< 0.05) with wasting (OR = 0.28 95% CI = 0.86,0.90) in the intervention group but not in the control group. In the control group the odds of being wasted were 2.43 times higher among children with diarrhea (OR = 2.43; 95% CI = 1.25,4.71; *p*-value = 0.009) compared with children who did not experience diarrhea (*p*-value> 0.05); no significant association was found in the intervention group. Furthermore, there was no significant association (*p*-value = 0.054) between seizures and wasting (OR = 4.88; 95% CI = 0.98,24.4). Fever in the two weeks before the interview date was not significantly associated with wasting (*p*-value = 0.339).

### The relationship between malnutrition and risk of infections

The households which were treating water at home before drinking it accounted for 28.05% of the total. Households that did not treat drinking water were highly likely (*p*-value< 0.05) to present stunted children. Being underweight was significantly associated (*p*-value< 0.05) with no water treatment at home, while there was no association between wasting and lack of water treatment.

Overall 86.98% of households had a toilet near the house (48.42% had a toilet within the compound of the house, 47.25% within a walking distance of 5 min; 3.63% between 5 to 10 min; 0.70% more than 10 min walking). Stunting was significantly associated with children who had no toilet near the house. Households with a toilet just outside the compound were less likely to present stunted (*p*-value< 0.05; OR = 0.62) and underweight (*p*-value< 0.05; OR = 0.53) children. Conversely, wasting was not associated with the presence or absence of toilet near the house. Own toilet was used in 3.63% of households during the day and in 3.2% of them at night, but there was no significant association between using private toilet during the day or at night and any outcome of malnutrition. Furthermore, there was no significant association between habits of washing utensils for feeding babies separately and any outcome of malnutrition.

### Association between malnutrition and control variables

From our data it emerged that children with birth weight greater than 4.2 Kg were less likely to become stunted (*p*-value = 0.005; OR = 0.32; 95% CI = 0.15,0.71) and underweight (*p*-value = 0.03; OR = 0.05; 95% CI = 0.006,0.34) in the first year of life, while LBW children were more likely to be stunted (*p*-value< 0.05; OR 2.43; 95% CI = 1.77, 3.32) and underweight (*p*-value< 0.05; OR 3.57; 95% CI = 2.56, 4.98). There was no significant association between birth weight and wasting.

Fully immunized children were less likely to be stunted (*p*-value< 0.05; OR = 0.70; 95% CI = 0.57, 0.85), but there was no significant association between wasted or underweight children and vaccination status.

There was a significant association (*p*-value< 0.05) between moderate and severe food insecurity and both stunting and underweight, but not with wasting.

Mothers married or with a partner were less likely to have stunted children (*p*-value< 0.05; OR = 0.75). From the bivariate analysis, no significant difference in the outcome of wasting between males and females emerged, but males were more likely to be stunted (*p*-value = 0.00) and underweight (*p*-value = 0.003).

### Multivariate regression analysis of determinants of nutritional status

According to the logistic regression model (see Tables 1, 2 and 3), male sex was significantly associated with stunting. Furthermore, shared toilets had a slightly significant association with stunting. Underweight was significantly associated with the age of the mother (children of mothers aged between 21 to 24 years were less likely to be underweight). Wasting had no significant association with the considered control variables.

**Table 1:** Determinants of wasting

Variables	Adjusted Odds Ratio	95% CI		*p*-value
		Lower limit	Upper limit	
Child’s age in months	0,61	0,27	1,37	0,23
Gender (Ref: Female)				
Male	4,02	0,12	137,18	0,44
Occurrence of illness in last 2 weeks (Ref:No)				
Yes	0,23	0,00	81,94	0,67
Child had Diarrhoea in the last two weeks (Ref:No)				
Yes	14,94	0,02	11,770,03	0,42
Utensils for feeding the baby washed separately (Ref: Yes)				
No	0,23	0,00	47,35	0,59
Birth weight (Ref: Birth weight > = 2500 g				
Birth weight < 2500 g	6,17	0,15	259,51	0,34
Exclusive breastfeeding at 6 months (Ref: No)				
Yes	1,13	0,09	15,38	0,93
Children who consumed at least 4 food groups (Ref: No)				
Yes	0,26	0,01	6,09	0,41
Study group (Ref: Control)				
Intervention	0,82	0,06	12,09	0,88
Constant	28,37	8,08E-06	9,96E+ 07	0,66

**Table 2:** Determinants of stunting

Variables	Adjusted Odds Ratio	95% CI		*p*-value
		Lower limit	Upper limit	
Occurrence of symptoms in last 2 weeks (Ref:No)				
Yes	0,31	0,06	1,62	0,17
Child had Cough + Rapid Breath in the last 2 weeks (Ref:No)				
Yes	6,32	0,18	226,66	0,31
Treating of drinking water (Ref:Yes)				
No	0,48	0,09	2,68	0,41
Handwashing behaviour (Ref: Never/Rarely/Sometimes)				
Often/Always	0,52	0,12	2,23	0,38
Utensils for feeding the baby washed separately (Ref:Yes)				
No	0,2	0,03	1,48	0,11
Location of toilet facility (Ref: Within the compound)				
Outside the compound	0,995	0,22	4,48	0,996
Toilet facility used at night (Ref: Own flush/traditional pit/VIP toilet)				
Shared flush/traditional pit/VIP toilet or other	0,02	0,00	1,09	0,055
Birth weight (Ref: Birth weight > = 2500 g				
Birth weight < 2500 g	52,92	2,83	989,93	0,008
Exclusive breastfeeding at 6 months (Ref: No)				
Yes	102,94	1,13	9409,22	0,044
Fully immunized children (Ref: No)				
Yes	0,77	0,19	3,16	0,72
Mothers occupation (Ref: Not employed)				
Housewife	0,41	0,06	2,97	0,38
Employed (salaried)	0,22	0,005	8,94	0,42
Self employed	7,61	0,70	82,21	0,095
Casual labourer	0,73	0,03	17,25	0,84
Mothers’ marital status (Ref: Not in a union)				
In a union	0,98	0,12	7,94	0,98
Age of mother/caregiver (years) (Ref: [14–20])				
21–24	2,51	0,42	14,96	0,31
25–29	1,79	0,237,095	13,56	0,57
30–45	1,40	0,126,400	15,39	0,79
Mothers’ educational attainment (Ref: Less than primary)				
Primary School	0,19	0,03	1,23	0,086
Secondary School	5,52	0,19	159,00	0,32
College/University	4,97	0,07	381,37	0,47
Number of children mother has (Ref: [1] child)				
2 children	0,53	0,04	7,73	0,65
3 children	0,14	0,01	3,40	0,23
Housefold food security (Ref: Food secure)				
Mild food insecure access	0,57	0,04	8,09	0,68
Moderate food insecure access	1,31	0,20	8,66	0,78
Severe food insecure access	0,74	0,09	6,03	0,78
Timely introduction of solid,semi-solid or soft foods (Ref: No)				
Yes	0,10	0,00	4,24	0,23
Children who consumed at least 4 food groups (Ref: No)				
Yes	0,04	0,005	0,31	0,002
Study group (Ref: Control)				
Intervention	0,59	0,12	2,77	0,50
Child’s age in months	0,81	0,54	1,22	0,32
Gender (Ref: Female)				
Male	4,99	1,04	24,09	0,045
Constant	23,081,97	0,11	4,69E+ 09	0,11

**Table 3:** Determinants of underweight

Variables	Adjusted Odds Ratio	95% CI		*p*- value
		Lower limit	Upper limit	
Child’s age in months	1,48	0,90	2,45	0,12
Gender (Ref: Female)				
Male	4,21	0,77	23,06	0,098
Child had fever in last 2 weeks (Ref:No)				
Yes	0,35	0,04	3,16	0,35
Child had Cough + Rapid Breath in the last 2 weeks (Ref:No)				
Yes	17,36	0,18	1689,60	0,22
Treating of drinking water (Ref:Yes)				
No	0,74	0,10	5,52	0,77
Handwashing behaviour (Ref: Never/Rarely/Sometimes)				
Often/Always	0,14	0,02	1,25	0,08
Utensils for feeding the baby washed separately (Ref:Yes)				
No	0,09	0,01	1,01	0,05
Location of toilet facility (Ref: Within the compound)				
Outside the compound	0,27	0,05	1,53	0,14
Birth weight (Ref: Birth weight > = 2500 g)				
Birth weight < 2500 g	2,26	0,28	18,39	0,45
Fully immunized children (Ref: No)				
Yes	0,76	0,15	3,74	0,74
Mothers occupation (Ref: Not employed)				
Housewife	0,08	0,01	0,97	0,047
Employed (salaried)	0,70	0,02	27,69	0,85
Self employed	0,22	0,01	4,87	0,34
Casual labourer	0,46	0,03	6,72	0,57
Mothers’ marital status (Ref: Not in a union)				
In a union	0,29	0,03	3,44	0,33
Mothers’ educational attainment (Ref: Less than primary)				
Primary School	0,85	0,09	8,45	0,89
Secondary School	3,82	0,12	125,21	0,45
College/University	0,69	0,01	53,94	0,87
Number of children mother has (Ref: [1] child)				
2 children	0,93	0,04	24,39	0,97
3 children	3,39	0,01	116,81	0,499
Age of mother/caregiver (years) (Ref: [14–20])				
21–24	0,07	0,01	0,78	0,03
25–29	0,08	0,01	0,95	0,045
30–45	0,20	0,02	2,83	0,23
Housefold food security (Ref: Food secure)				
Mild food insecure access	1,10	0,04	28,71	0,95
Moderate food insecure access	7,51	0,75	75,48	0,09
Severe food insecure access	1,95	0,15	25,63	0,61
Minimun meal frequency (Ref: No)				
Yes	1,19	0,13	10,91	0,88
Study group (Ref: Control)				
Intervention	0,21	0,043	1,21	0,08
Constant	26,53	0,00	309,488,30	0,49

## Discussion

The prevalence of undernutrition was consistent with national data, where stunting among children < 5 years of age is estimated to be 26% - and even higher when considering wasting (affecting 4% of all Kenyans) [31]. Moreover, we considered only the first year of life, while stunting predominantly occurs in the first 1000 days of life (0–23 months) and then continues until the age of five. Our finding of a higher prevalence of wasting between birth and 6 months of age challenges the belief that malnutrition largely begins during the period of complementary feeding, between 6 and 24 months of age.

Literature indicates that wasting (weight for age z score, WHZ) and stunting (height for age z score, HAZ) have many common risk factors suggesting that both conditions might have common causes, although different strengths of causality have been identified; while wasting is usually due to acute nutrient deficiency, stunting is indicative of chronic malnutrition. From our data, wasting was significantly associated with common childhood illnesses probably due to the severe loss of weight that can result from an acute episode of infection in infants.

The relationship between common acute infections and stunting is difficult to explore, especially in the slums, where multiple factors may concur at the same time. For example, documented indoor air pollution caused by solid fuels such as firewood is strongly associated with increased risk of respiratory tract infections [32]. Moreover, catch-up growth may be seen between acute infectious episodes, only if an adequate nutritional intake is provided and the interval between infections is long enough [33, 34]. On one hand, when the infectious episodes are frequent and nutritional intake is not adequate, catch-up is not possible and diarrhea can persist associated with malabsorption. On the other hand, children who live in poor hygienic conditions become stunted because they present multiple acute episodes of gastroenteritis as a result of chronic infectious diarrhea and specific pathogens may be involved [5]. Therefore, gut damage appears to arise early in infancy (from around 3 to 6 months of age), because in many developing countries complementary foods are commonly introduced as early as 2 months of age, often with low nutritional value and the inherent risks of microbial contamination [35, 36].

In our analysis, stunting is associated with the WASH situation. The results of our study in the slums demonstrate a significant association between inadequate access to safe drinking water and stunting. This could be attributed to the fact that clean water can prevent the spread of water-borne diseases which can negatively affect the health and nutrition status of young children. Our findings also reinforce earlier evidence that WASH-related conditions are significantly associated with stunting. Moreover, the significant association of stunting with toilet distance might be an indirect determinant of poverty and not only a correlation with the hygiene situation.

Among other feeding practices, based on our data high food insecurity might be connected to the specific environment of the slums.

Concerning factors related to morbidity and nutritional status, we also evaluated immunization coverage as a protective factor for both. Within our population stunted children were less likely to complete the immunization program. Vaccination adherence could also be an indirect sign of parent’s awareness of babies’ health and care, which may result in a greater protection against malnutrition.

Among the chosen control variables, which are similar to other studies from Africa [37], the current study observed LBW as an independent risk factor for children’s wasting. Evidence shows that undernourished mothers often deliver LBW infants, which is an independent risk factor for childhood malnutrition, morbidity, and mortality [38].

Furthermore, the association with socioeconomic determinants is weaker in wasted than in stunted children, as found in other studies [39]. For example, there was no difference in wasting between male and female in the bivariate analysis but there was a significant association with male sex when we considered stunting and underweight. From the logistic regression model males emerged as being more likely to be stunted. Other evidence from Kenya showed that there might be a strong gender inequality in intra-household food distribution, leading to more girls experiencing stunting, wasting and infectious diseases [40]; however, in different studies male were actually more likely to be stunted [41]. The care of children is often closely linked with cultural and gender issues that may be influenced in different ways, according to specific environments; this is true in the targeted setting as well, where determinants appear different to those characterizing rural areas.

### Limitations of the study

It is important to acknowledge that, given the secondary data sources used for this analysis, some limitations call for a prudent consideration of the conclusion reached. As the study was questionnaire-based, predetermined sets of variables were selected and questions that required a good memory were vulnerable to recall bias. Moreover, most of the data on morbidities were collected through reporting by the primary caregiver, without a clinical confirmation - a feature which might explain the heterogeneity of the reported symptoms.

## Conclusions

In the specific environment where the study was conducted acute malnutrition is correlated with acute infections, while chronic malnutrition is more influenced by WASH conditions. So, our findings suggest that one cannot separate infection and its risk factors as determinants of the whole malnutrition burden. Furthermore, similar determinants of malnutrition and infections are significantly present in the slums and they may adversely impact children’s health and development.

Data and information obtained through this and other prospective studies can be used for planning distribution and utilization of available healthcare resources, as well as for developing effective disease control measures.

## Abbreviations

APHRC: African Population and Health Research Center
BW: Birth Weight
CHVs: Community Health Volunteers
CI: Confidence Interval
CUs: Community Units
EBF: Exclusive Breastfeeding
EE: Enteropathy
HAZ: Height for Age Z score
HFIAS: Household Food Insecurity Access Scale
LBW: Low Birth Weight
MYICN: Maternal Infant and Young Child Nutrition
NUHDSS: Nairobi Urban Health and Demographic Surveillance System
OR: Odds Ratio
WASH: Water, Sanitation and Hygiene
WAZ: Weight for Age Z score
WHO: World Health Organization
WHZ: Weight for Height Z score
